# A prognostic model based on immune-related long noncoding RNAs for patients with epithelial ovarian cancer

**DOI:** 10.1186/s13048-021-00930-w

**Published:** 2022-01-15

**Authors:** Yao Peng, Hui Wang, Qi Huang, Jingjing Wu, Mingjun Zhang

**Affiliations:** 1 Department of Oncology, The Second Affiliated Hospital of Anhui Medical University, No. 678, Furong Road, Hefei, 230601 Anhui P.R. China; 2 Anhui Medical University, No. 81, Meishan Road, Hefei, 230032 Anhui P.R. China; 3 Department of Oncology, Lu’an People’s Hospital of Anhui Province, No. 21, West Anhui Road, Lu’an, 237006 Anhui P.R. China

**Keywords:** Epithelial ovarian cancer, Immune, Long noncoding RNAs, Prognostic signature, TCGA

## Abstract

**Background:**

Long noncoding RNAs (lncRNAs) are important regulators of gene expression and can affect a variety of physiological processes. Recent studies have shown that immune-related lncRNAs play an important role in the tumour immune microenvironment and may have potential application value in the treatment and prognosis prediction of tumour patients. Epithelial ovarian cancer (EOC) is characterized by a high incidence and poor prognosis. However, there are few studies on immune-related lncRNAs in EOC. In this study, we focused on immune-related lncRNAs associated with survival in EOC.

**Methods:**

We downloaded mRNA data for EOC patients from The Cancer Genome Atlas (TCGA) database and mRNA data for normal ovarian tissue from the Genotype-Tissue Expression (GTEx) database and identified differentially expressed genes through differential expression analysis. Immune-related lncRNAs were obtained through intersection and coexpression analysis of differential genes and immune-related genes from the Immunology Database and Analysis Portal (ImmPort). Samples in the TCGA EOC cohort were randomly divided into a training set, validation set and combination set. In the training set, Cox regression analysis and LASSO regression were performed to construct an immune-related lncRNA signature. Kaplan–Meier survival analysis, time-dependent ROC curve analysis, Cox regression analysis and principal component analysis were performed for verification in the training set, validation set and combination set. Further studies of pathways and immune cell infiltration were conducted through Gene Set Enrichment Analysis (GSEA) and the Timer data portal.

**Results:**

An immune-related lncRNA signature was identified in EOC, which was composed of six immune-related lncRNAs (KRT7-AS, USP30-AS1, AC011445.1, AP005205.2, DNM3OS and AC027348.1). The signature was used to divide patients into high-risk and low-risk groups. The overall survival of the high-risk group was lower than that of the low-risk group and was verified to be robust in both the validation set and the combination set. The signature was confirmed to be an independent prognostic biomarker. Principal component analysis showed the different distribution patterns of high-risk and low-risk groups. This signature may be related to immune cell infiltration (mainly macrophages) and differential expression of immune checkpoint-related molecules (PD-1, PDL1, etc.).

**Conclusions:**

We identified and established a prognostic signature of immune-related lncRNAs in EOC, which will be of great value in predicting the prognosis of clinical patients and may provide a new perspective for immunological research and individualized treatment in EOC.

**Supplementary Information:**

The online version contains supplementary material available at 10.1186/s13048-021-00930-w.

## Introduction

Ovarian cancer (OC) is one of the most common gynaecological malignancies in the world, with a survival rate of less than 50% 5 years after diagnosis [1]. Epithelial ovarian cancer (EOC) is the most common subtype, accounting for more than 90% of OC [2]. Despite the rapid development of therapeutic approaches such as surgery, chemotherapy, targeted therapy and immunotherapy, most patients are diagnosed in advanced clinical stages with poor prognosis due to the lack of specific signs and symptoms at an early stage and effective clinical screening methods [3, 4]. At present, the first-line therapy for newly diagnosed EOC is complete cytoreductive surgery followed by platinum-based chemotherapy [5]. The sensitivity of first-line therapy is better in patients with stage I-IIA lesions confined to the ovary. At stage IIB-IV, cancer cells have metastasized to the peritoneum and show resistance to first-line treatment, with a very high recurrence rate [6, 7]. Therefore, to improve the survival rate of EOC patients, it is necessary to find new prognostic biomarkers and improve the prediction of prognosis.

Long noncoding RNAs (lncRNAs) are a kind of noncoding RNA with a length of more than 200 nucleotides. The expression levels of lncRNAs are relatively low in tissues, but they are widely distributed in various organs, such as the brain, lung, heart and ovary [8]. Despite the lack of protein coding function, lncRNAs are involved in various types of gene regulation, including epigenetic, transcriptional and posttranscriptional regulation. These regulations are closely related to the occurrence, development and prognosis of tumours and other diseases and play important physiological roles in tumour cell proliferation, apoptosis, metastasis, invasion and migration [9–11]. In recent years, increasing evidence has shown that the unregulated expression of lncRNAs is related to cancer. For example, the upregulation of lncRNA HOTAIR is associated with various cancers, such as breast cancer, colorectal cancer, liver cancer and oesophageal cancer [12–15]. Studies have reported that lncRNA PTAF promotes epithelial mesenchymal transformation (EMT) of OC by regulating SNAI2 expression through miR-25 and that lncRNA SPOCD1-AS promotes peritoneal metastasis of OC by interacting with G3BP1 to reshape mesenchymal cells [16, 17]. Increased research on lncRNAs will contribute to the understanding of tumour cell function and may lead to new clinical applications in oncology. One of the important characteristics of malignant tumours is the ability to escape immune surveillance, such as avoiding recognition by downregulating the expression of MHC-I molecules and forming an immunosuppressive tumour microenvironment (TME) to avoid killing [18]. Immune-related lncRNAs (IR-lncRs) play an important role in TME remodelling. They mediate immune activation and inhibit the immune response and perform their biological functions in a variety of ways, such as directly or indirectly affecting transcriptional regulation, regulating protein and mRNA stability, and via competitive endogenous networks [19, 20]. IR-lncRs are important regulators of immune cell-specific gene expression. Shang et al. found that the lncRNA HOTTIP can upregulate the expression of PD-L1 in neutrophils to enhance the expression of IL-6, thereby promoting the immune escape of OC cells [21, 22]. Currently, immunotherapy is widely used in clinical practice and can regulate the TME [23]. Although the benefits of immunotherapy are significant, there are still many patients with low sensitivity and high resistance to immunotherapy. Therefore, it is of great significance to develop new and more sensitive prognostic biomarkers and antitumour targets.

In this study, we obtained high-throughput sequencing data of EOC from The Cancer Genome Atlas (TCGA, https://portal.gdc.cancer.gov/, March 2021) data portal, then we identified and used the differential expression of IR-lncRs to build a signature to predict the prognosis of patients with EOC. In addition, we verified the signature. Finally, we further performed pathway enrichment analysis and immune infiltration function evaluation on the prediction signature.

## Materials and methods

### Data collection and identification of differentially expressed genes (DEGs)

We downloaded mRNA transcriptome data from 379 patients with EOC and clinicopathological information from 587 patients with EOC from the TCGA. At the same time, mRNA transcriptome data of 88 normal ovarian tissues from the Genotype-Tissue Expression (GTEx, https://www.gtexportal.org/home/index.html) database were included in the analysis. We normalized the mRNA data of 379 EOC patients and 88 normal ovarian tissues by fragment per million exon model. Through the Immunology Database and Analysis Portal (ImmPort, https://immport.niaid.nih.gov), resources related to immunology research can be collected, organized and shared, from which we obtained a list of 2483 immune-related genes (IRGs) (Attachment 1). The above data are available to the public, so they are not subject to review by an ethics committee. The mRNA data from the TCGA and GTEx portal were consolidated into a matrix file using Perl (https://www.perl.org/), and then differential expression analysis between EOC and normal tissue was performed using the “Limma package” in R (version 4.0.3) to identify the DEGs. The filter criteria were an FDR less than 0.05 and |log2(FC)| greater than 1. According to The Gene Coding Plan (https://www.gencodegenes.org/), lncRNA profiles were extracted from the mRNA expression profiles of the TCGA.

### Mining differentially expressed IRGs and IR-lncRs

The differentially expressed IRGs (DE-IRGs) were extracted from the overlap of IRGs and DEGs. |COR| > 0.4 and *P* < 0.001 were set as the cut-off value, the R package “Limma” was used to analyse the coexpression of DE-IRGs and lncRNAs in the TCGA, and the differentially expressed IR-lncRs were obtained for subsequent analysis.

### Construction of a prognostic model based on differential expression of IR-lncRs

After excluding patients with incomplete prognostic information, 374 EOC patients with complete overall survival (OS) information were finally included. They were randomly divided into a training set (*n* = 236) and a validation set (*n* = 138) at a 5:3 ratio, and all patients were regarded as a combination set (*n* = 374). The IR-lncR prediction model was constructed with the data from the training set and verified in the validation set and the combination set. First, univariate Cox regression analysis was performed on the training set to screen out IR-lncRs that were significantly correlated with OS (*P* < 0.05). Next, the least absolute shrinkage and selection operator (LASSO) regression model was used to perform a multivariate Cox proportional hazard regression analysis. After 1000 cross-validations, the lambda value with the smallest error was finally determined, and the model was refitted with the best lambda value. Using multivariate Cox regression to establish a prognostic risk assessment model, we obtained the risk score calculation formula as follows: Risk Score = lncRNA1 expression value × β1 + lncRNA2 expression value × β2 + … … + lncRNAn expression value × βn. Where β represented the regression coefficient calculated by the multivariate Cox regression model. EOC patients in the training, validation and combination sets were divided into high-risk and low-risk groups according to the median risk score. Finally, the R packages “Pheatmap”, “SurvMiner”, “Survival” and “Survivalroc” were used to evaluate the accuracy of the model in the training, validation and combination sets, respectively. We drew a risk heatmap, risk curve and survival state chart. The Kaplan–Meier method was used to generate survival curves for the high-risk and low-risk groups. Time-dependent receiver operating characteristic (ROC) curves were drawn, and the area under the curve (AUC) was calculated at 3 years and 5 years.

### Independent prognostic analysis and construction of the nomogram

To determine the predictive effect of the constructed IR-lncR model on prognosis, we excluded patients who lacked detailed clinicopathological information, including age, histological grade and FIGO stage. We used the R packages “Survival” and “Forestplot” to incorporate risk scores and clinical indicators into univariate and multivariate Cox regression analyses. We used the “Rms” package to construct a nomogram to predict 1-, 3-, and 5-year survival in patients with EOC in conjunction with risk scores and clinical indicators. The calibration curve of the nomogram was used to evaluate the accuracy of the prediction effect. We also used ROC curves to compare a nomogram containing only one independent prognostic factor with a nomogram containing all independent prognostic factors. In addition, the R package “Vioplot” was used to visualize the expression of each lncRNA in the signature between normal ovarian tissue and OC tissue, and the R package “corrplot” and Pearson correlation test were used to illustrate the interactions between each lncRNA.

### Gene set enrichment analysis (GSEA)

GSEA (http://software.broadinstitute.org/gsea/index.jsp) was used to understand the expression status of certain genes in specific functional gene sets. According to the prediction model, the TCGA EOC samples were divided into high-risk and low-risk groups. C7.all.v7.3.Symbols.GMT from the Molecular Signature Database (MSigDB, https://www.gsea-msigdb.org/gsea/msigdb/index.jsp) was selected as the reference gene bank. GSEA was used to identify differences in biological function between the two groups.

### Evaluation of immune status and immune cell infiltration based on a predictive model

The R packages “Limma” and “Scatterplot3D” were used to perform principal component analysis (PCA) on the gene expression profiles of the TCGA EOC cohort, and the immune status and expression pattern of the subgroup samples were visualized. The Tumour Immune Estimation Resource (Timer, https://cistrome.shinyapps.io/timer/) data portal can be used to systematically assess the impact of different immune cells on cancer. We downloaded the level of immune cell infiltration in EOC patients from the Timer to evaluate the correlation between IR-lncRs and immune cell infiltration. At the same time, to clearly show the distribution of immune checkpoint-related gene expression in the prediction model, we used the R packages “Limma” and “Beeswarm” to draw the box map and analysed the differential expression of eight immune checkpoint-related genes between the low-risk and high-risk groups.

## Results

**Fig. 1:**
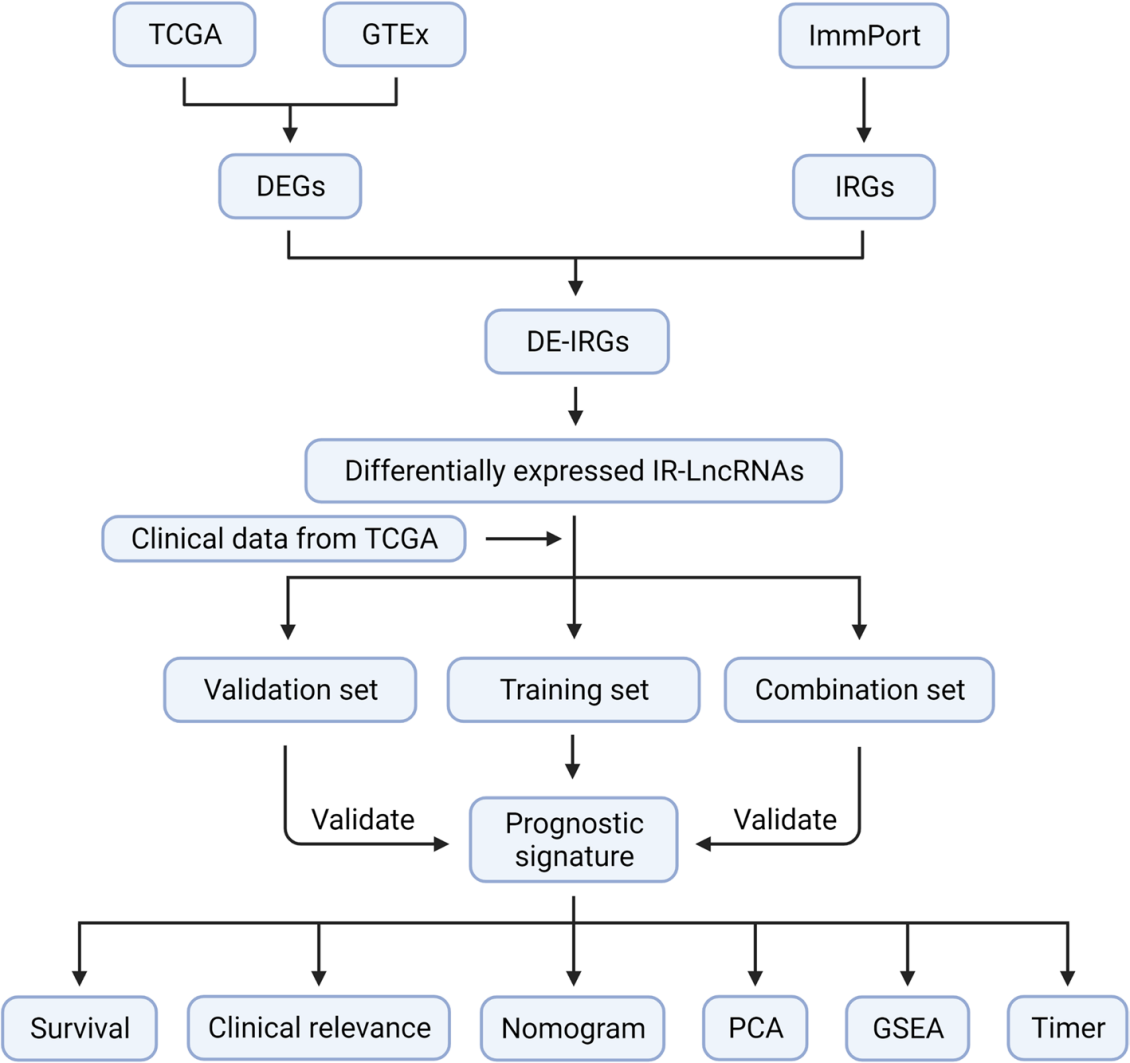
The flow diagram for the entire study

This research was carried out according to the procedure shown in Fig. 1.

### Identification of DE-IRGs and IR-IncRs in EOC

**Fig. 2:**
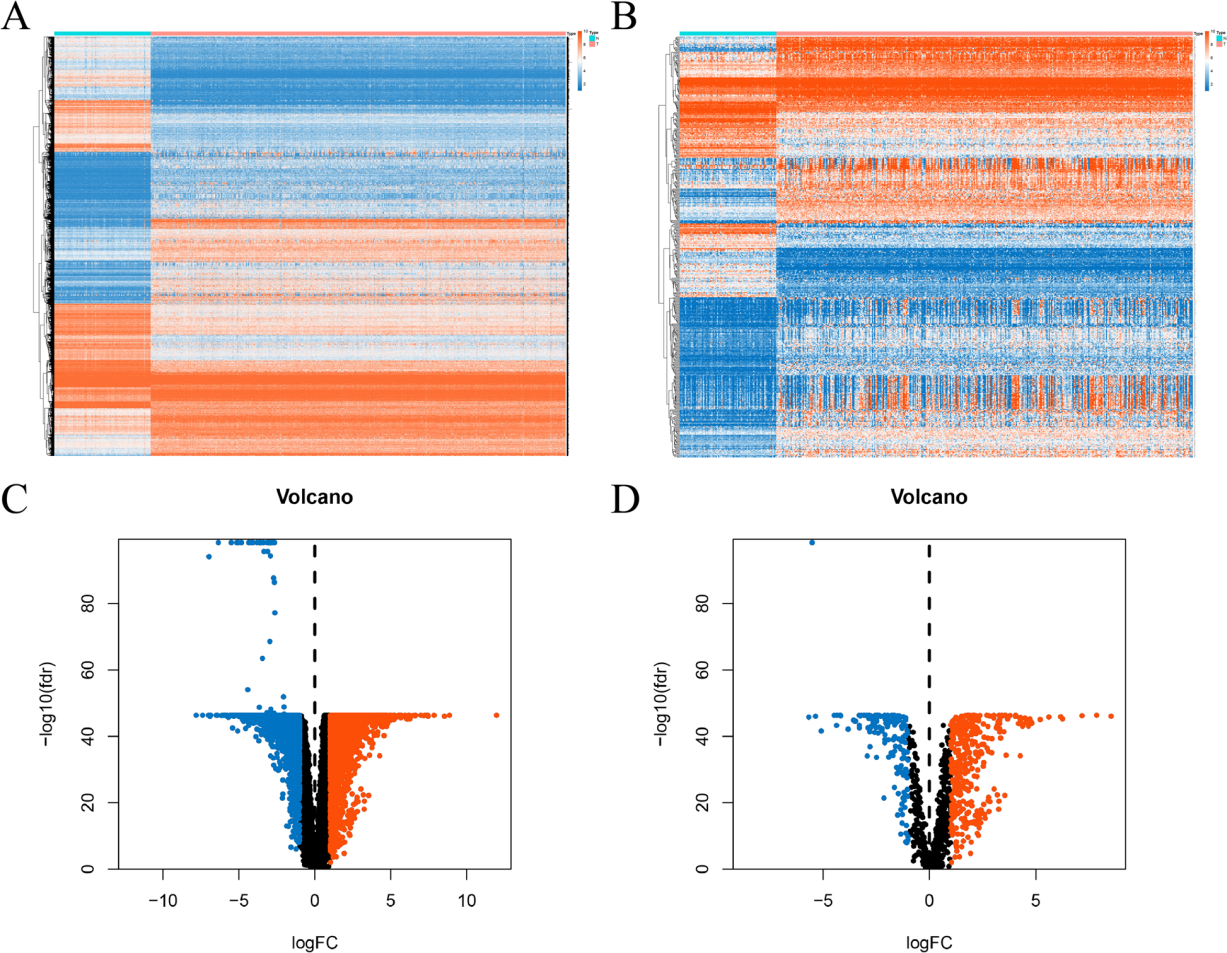
DE-IRGs were identified from 379 cases of ovarian cancer and 88 cases of normal ovarian tissue. **A**, **C** Heatmap and volcano of the DEGs in EOC. **B**, **D** Heatmap and volcano of the DE-IRGs in EOC

We identified a total of 7255 DEGs between tumours and normal tissues, including 3790 upregulated genes and 3465 downregulated genes (Fig. 2A and C). By intersecting these DEGs with the 2483 IRGs from ImmPort, we found 339 upregulated DE-IRGs and 157 downregulated DE-IRGs in EOC (Fig. 2B and D). Through common expression analysis, we identified 421 differentially expressed IR-lncRs (|COR| > 0.4, *p* < 0.001) (Attachment 2).

### Construction of an IR-lncR signature in the TCGA EOC cohort

**Fig. 3:**
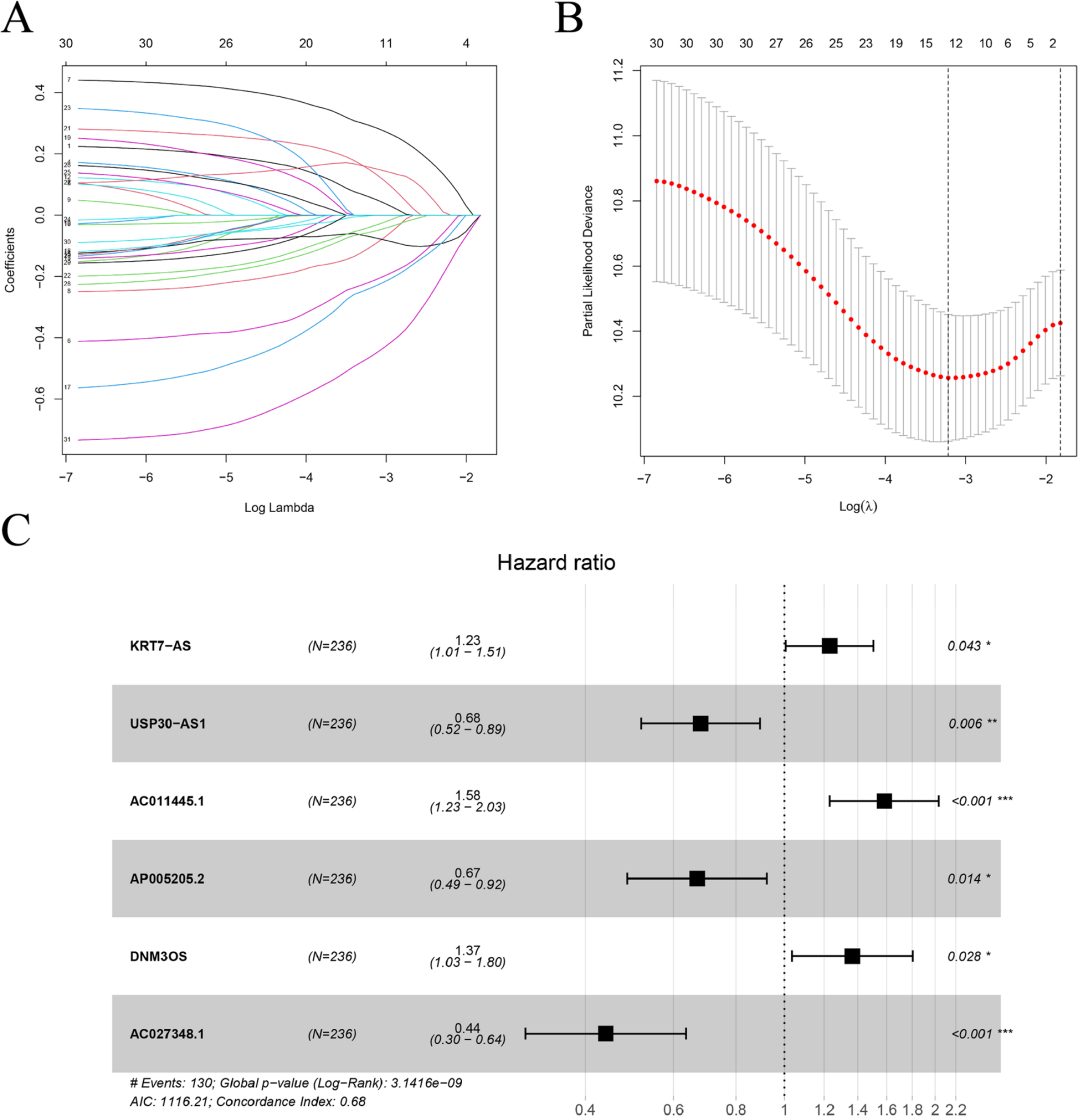
Construction of an immune-related lncRNA prognostic model based on the training set. **A** LASSO coefficient profiles of the 32 candidates in the training set. **B** A plot of thousand-fold cross-validation error rates. Selection of the optimal parameter (lambda) in the LASSO model. **C** Forest plot of six candidate immune-related lncRNAs associated with the survival of EOC were screened by multivariate Cox regression analysis

A total of 374 EOC patients with complete OS information were included from the TCGA for the follow-up study. To increase the credibility of the study, we divided the whole dataset into a training set and a verification set and defined the whole dataset as a combination set. The expression profiles of 421 IR-lncRs in the training set were used to construct a prognostic prediction model. Univariate Cox regression analysis was performed on the expression profiles of lncRNAs in the training set, and 32 IR-lncRs were significantly correlated with OS (*P* < 0.05) (Table 1). LASSO regression was performed on these IR-lncRs to prevent overfitting of the model, and the prediction accuracy was estimated through 1000 cross validations (Fig. 3A-B). Next, we identified six key IR-lncRs using multivariate Cox regression analysis (Fig. 3C). These six IR-lncRs were used in the prognostic model construction, and they were KRT7-AS, USP30-AS1, AC011445.1, AP005205.2, DNM3OS and AC027348.1, and the corresponding coefficients were also given (Table 2). The final risk score calculation formula was as follows: Risk score = expression value of KRT7-AS * 0.2079 + expression value of USP30-AS1 * (− 0.3862) + expression value of AC011445.1 * 0.4593 + expression value of AP005205.2 * (− 0.4020) + expression value of DNM3OS * (0.3120) + expression value of AC027348.1 * (− 0.8224).

**Table 1:** Univariate Cox analysis of immune-related lncRNAs

Id	HR	HR.95 L	HR.95H	*P*-value
AC040169.1	0.626480	0.480216	0.817292	0.000566
AC011445.1	1.472847	1.155252	1.877753	0.001781
AC027348.1	0.605601	0.439290	0.834877	0.002201
AP005205.2	0.638080	0.468680	0.868708	0.004317
AC010531.6	0.656519	0.489907	0.879795	0.004841
AC083880.1	0.630212	0.456412	0.870194	0.005039
UBXN10-AS1	0.713760	0.562469	0.905743	0.005528
HCG14	0.655622	0.483959	0.888175	0.006419
AC091153.3	0.663274	0.485976	0.905257	0.009676
AC020916.2	1.530921	1.104282	2.122391	0.010615
USP30-AS1	0.737146	0.576721	0.942196	0.014873
AC135050.6	0.685481	0.503844	0.932598	0.016207
CDC37L1-DT	0.702602	0.523147	0.943614	0.018993
AC073046.1	1.358341	1.050206	1.756884	0.019640
LINC02004	0.688872	0.500912	0.947360	0.021871
KMT2E-AS1	0.735848	0.564351	0.959461	0.023473
KRT7-AS	1.238102	1.025378	1.494957	0.026384
ATP2A1-AS1	0.742762	0.569485	0.968762	0.028227
AL137802.2	0.680427	0.481615	0.961309	0.028980
AL035701.1	0.687185	0.489170	0.965355	0.030519
TGFB2-AS1	0.793979	0.643097	0.980262	0.031924
DLG3-AS1	0.722241	0.533965	0.976904	0.034721
AL451165.2	0.774204	0.608127	0.985636	0.037764
DNM3OS	1.341397	1.014270	1.774030	0.039465
AP001453.2	0.785556	0.623085	0.990391	0.041188
AC010326.3	0.719230	0.523362	0.988402	0.042165
PCAT6	0.823436	0.681595	0.994794	0.044001
AL391069.2	0.787554	0.624064	0.993875	0.044250
U62317.1	0.710871	0.509250	0.992319	0.044934
AC133552.5	0.792207	0.630022	0.996142	0.046257
AC103769.1	0.757677	0.574379	0.999470	0.049563

**Table 2:** Multiple Cox analysis of EOC-specific immune-related lncRNAs

Id	Coef	HR	HR.95 L	HR.95H	*P*-value
KRT7-AS	0.20788	1.231066	1.006212	1.506167	0.043368
USP30-AS1	−0.38621	0.679631	0.516830	0.893714	0.005705
AC011445.1	0.45933	1.583010	1.231711	2.034504	0.000333
AP005205.2	−0.40195	0.669012	0.485082	0.922683	0.014264
DNM3OS	0.31196	1.366101	1.034135	1.804631	0.028073
AC027348.1	−0.82237	0.439388	0.303846	0.635395	0.000012

### Verification of the 6-lncRNA signature for survival prediction

**Fig. 4:**
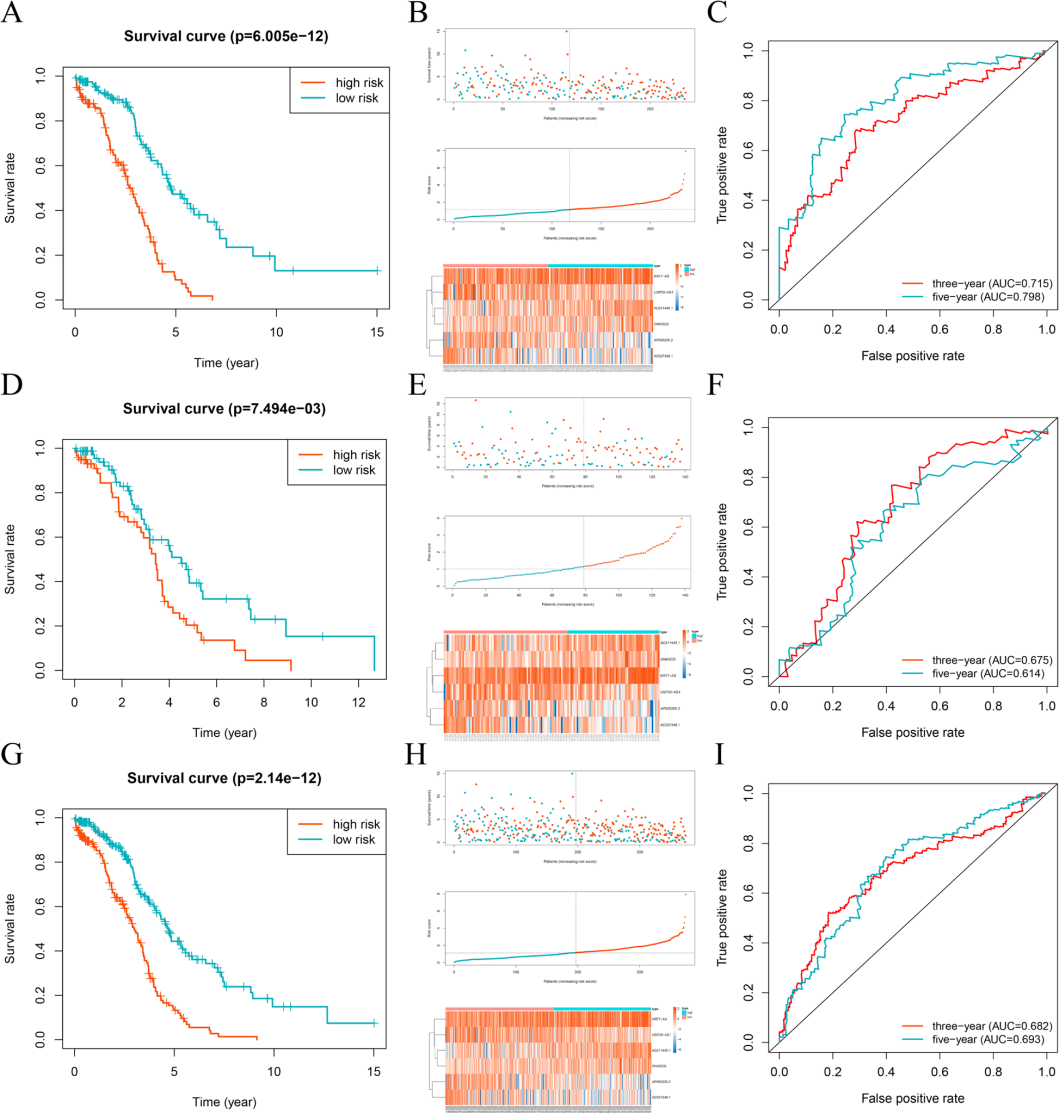
Verification of survival prediction ability and analysis of the risk score of the 6-lncRNA signature in EOC. **A**, **B**, **C** Kaplan–Meier curve, survival state chart, risk curve, heatmap of lncRNA expression and time-dependent ROC curve of the 6-lncRNA signature in the training set. **D**, **E**, **F** Kaplan–Meier curve, survival state chart, risk curve, heatmap of lncRNA expression and time-dependent ROC curve of the 6-lncRNA signature in the validation set. **G**, **H**, **I** Kaplan–Meier curve, survival state chart, risk curve, heatmap of lncRNA expression and time-dependent ROC curve of the 6-lncRNA signature in the combination set

We constructed and verified a good 6-lncRNA signature for survival prediction. The training set risk scores were calculated according to the risk score calculation formula and the lncRNA expression profiles of the TCGA EOC cohort. Then, using the median risk score as a cut-off point, patients were divided into high-risk and low-risk groups. In the training set, the mortality of patients increased with an increase in risk score (Fig. 4A). The survival status of the low-risk group was better than that of the high-risk group, the OS of the low-risk group was significantly higher than that of the high-risk group, and the heatmap showed the expression of 6 lncRNAs in the training set (Fig. 4B). The AUC values of 3-year and 5-year OS were 0.715 and 0.798, respectively (Fig. 4C). To evaluate the accuracy of the prediction model, it was validated in the validation set and the combination set. Patients in the validation and combination sets were also divided into low-risk and high-risk groups based on the median risk score of the training set. The results showed that the mortality of patients in both the validation and combination sets increased with increasing risk score (Fig. 4D and G). The survival status of the low-risk group was better than that of the high-risk group, the OS of the low-risk group was higher than that of the high-risk group, and the expression of 6 lncRNAs in the validation and combination sets was also shown by a heatmap (Fig. 4E and H). In the validation set, the AUC values of 3-year and 5-year OS were 0.675 and 0.614, respectively (Fig. 4F). In the combination set, the AUC values of 3-year and 5-year OS were 0.682 and 0.693, respectively (Fig. 4I).

### Predictive model as an independent prognostic factor evaluation

We included clinicopathological features such as age, histological grade and FIGO stage, as well as risk score in the analysis. Univariate and multivariate Cox regression analyses were used to determine whether the 6-lncRNA signature was an independent prognostic factor. Univariate Cox analysis results showed that age and risk score were independent prognostic factors for EOC patients in the training and combination sets, while only risk score was an independent prognostic factor in the validation set. The results of multivariate Cox analysis also indicated that the predictive model was a reliable independent prognostic indicator in the training, validation and combination sets (Table 3).

**Table 3:** Univariate and multivariate Cox analysis of the clinical features of EOC patients in each set

Variables	Univariate COX analysis		P	Multivariate COX analysis		P
	HR	95%CI		HR	95%CI	
TCGA training set						
Age (≤55 vs > 55)	1.52	1.03–2.25	0.04	1.39	0.93–2.06	0.11
Grade (G1 vs G2 vs G3 vs G4)	1.24	0.78–1.97	0.37	1.04	0.65–1.68	0.86
Stage (Stage I vs Stage II vs Stage III vs Stage IV)	1.46	0.97–2.21	0.07	1.44	0.93–2.23	0.10
Riskscore (high/low)	1.70	1.49–1.93	0.00	1.68	1.47–1.92	0.00
TCGA verification set						
Age (≤55 vs > 55)	1.14	0.69–1.88	0.61	1.16	0.70–1.93	0.56
Grade (G1 vs G2 vs G3 vs G4)	2.09	0.84–5.20	0.11	2.08	0.83–5.22	0.12
Stage (Stage I vs Stage II vs Stage III vs Stage IV)	1.21	0.76–1.92	0.43	1.13	0.68–1.87	0.63
Riskscore (high/low)	1.47	1.14–1.89	0.00	1.47	1.15–1.88	0.00
TCGA combination set						
Age (≤55 vs > 55)	1.36	1.00–1.85	0.05	1.32	0.97–1.79	0.08
Grade (G1 vs G2 vs G3 vs G4)	1.39	0.92–2.09	0.12	1.25	0.82–1.88	0.30
Stage (Stage I vs Stage II vs Stage III vs Stage IV)	1.33	0.98–1.80	0.07	1.29	0.94–1.77	0.11
Riskscore (high/low)	1.63	1.45–1.83	0.00	1.61	1.43–1.81	0.00

### Construction and verification of the nomogram

**Fig. 5:**
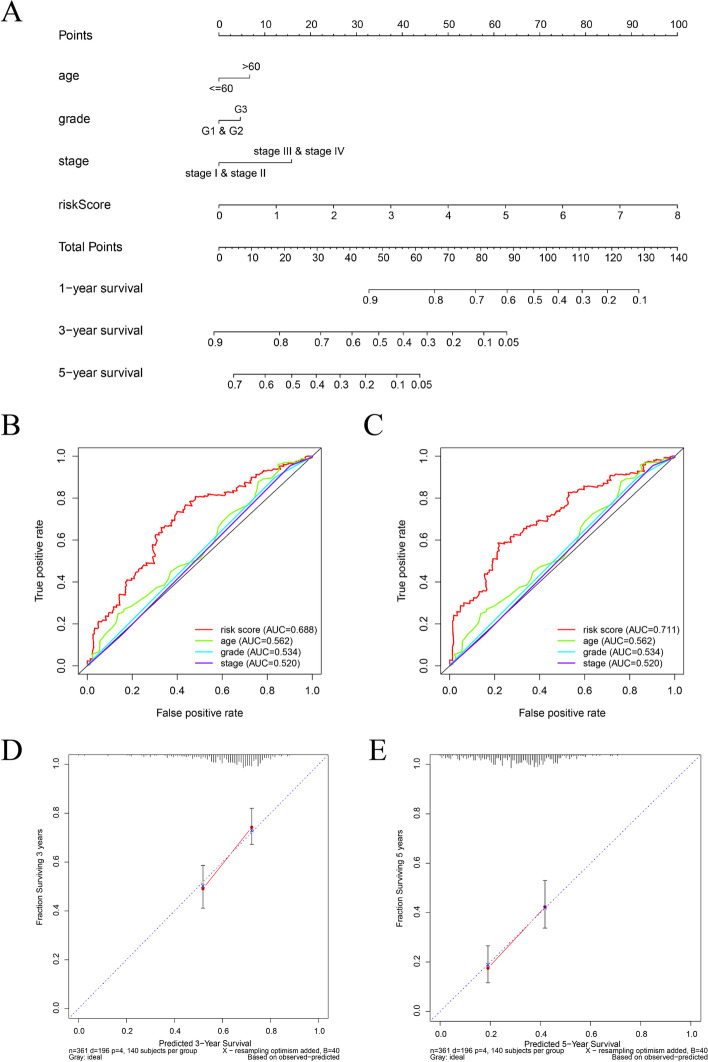
Nomogram for predicting the overall survival probability of EOC patients. **A** The nomogram was built based on age, histological grade, FIGO stage and risk score. **B**, **C** The time-dependent ROC curve of the nomogram for 3- and 5-year overall survival in EOC patients. **D**, **E** The calibration curves for internal validation of the nomogram at 3 and 5 years

We developed a nomogram for predicting survival risk in EOC patients based on the entire TCGA EOC cohort. Prognostic indicators such as age, grade, stage and risk score were included in the nomogram (Fig. 5A). The 3-year and 5-year OS of the nomogram were 0.688 and 0.711, respectively, which were significantly more valuable than a single clinicopathological index (Fig. 5B-C). In addition, the calibration chart showed a high consistency between the prediction and actual observation of the 3-year and 5-year survival rates for EOC patients (Fig. 5D-E).

### Evaluation of immune infiltrate function in low-risk and high-risk populations

**Fig. 6:**
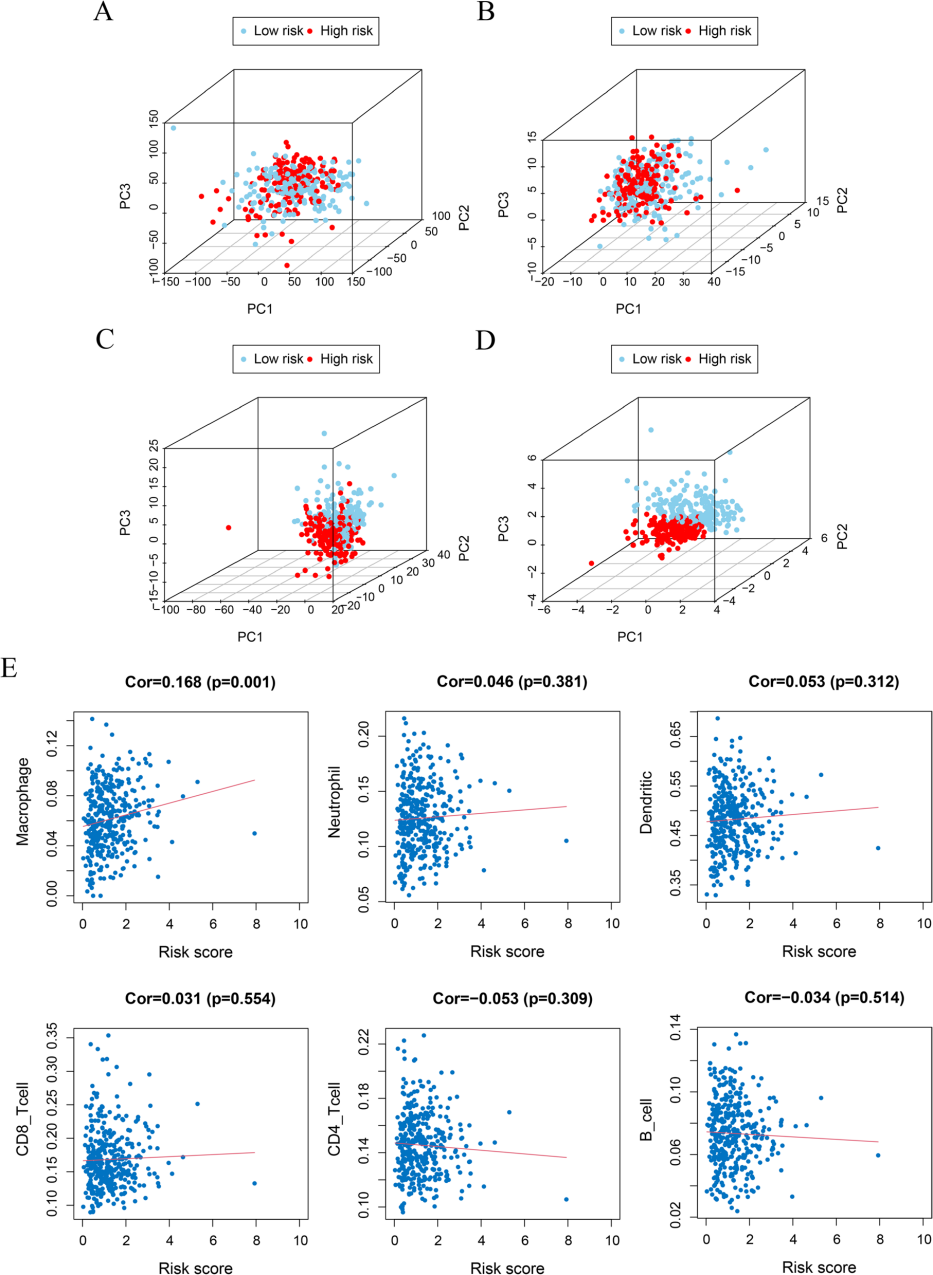
The immune status and immune cell infiltration in the high-risk and low-risk groups were evaluated by principal component analysis (PCA) and Tumour Immune Estimation Resource (Timer). **A** PCA map based on genome-wide expression set. **B** PCA map based on the immune-related gene expression set. **C** PCA map based on immune-related lncRNA set. **D** PCA map based on the 6-lncRNA signature. **E** The relationship between the risk score and infiltration abundance of six kinds of immune cells was analysed based on the 6-lncRNA signature: macrophages, neutrophils, dendritic cells, CD8 T cells, CD4 T cells and B cells

**Fig. 7:**
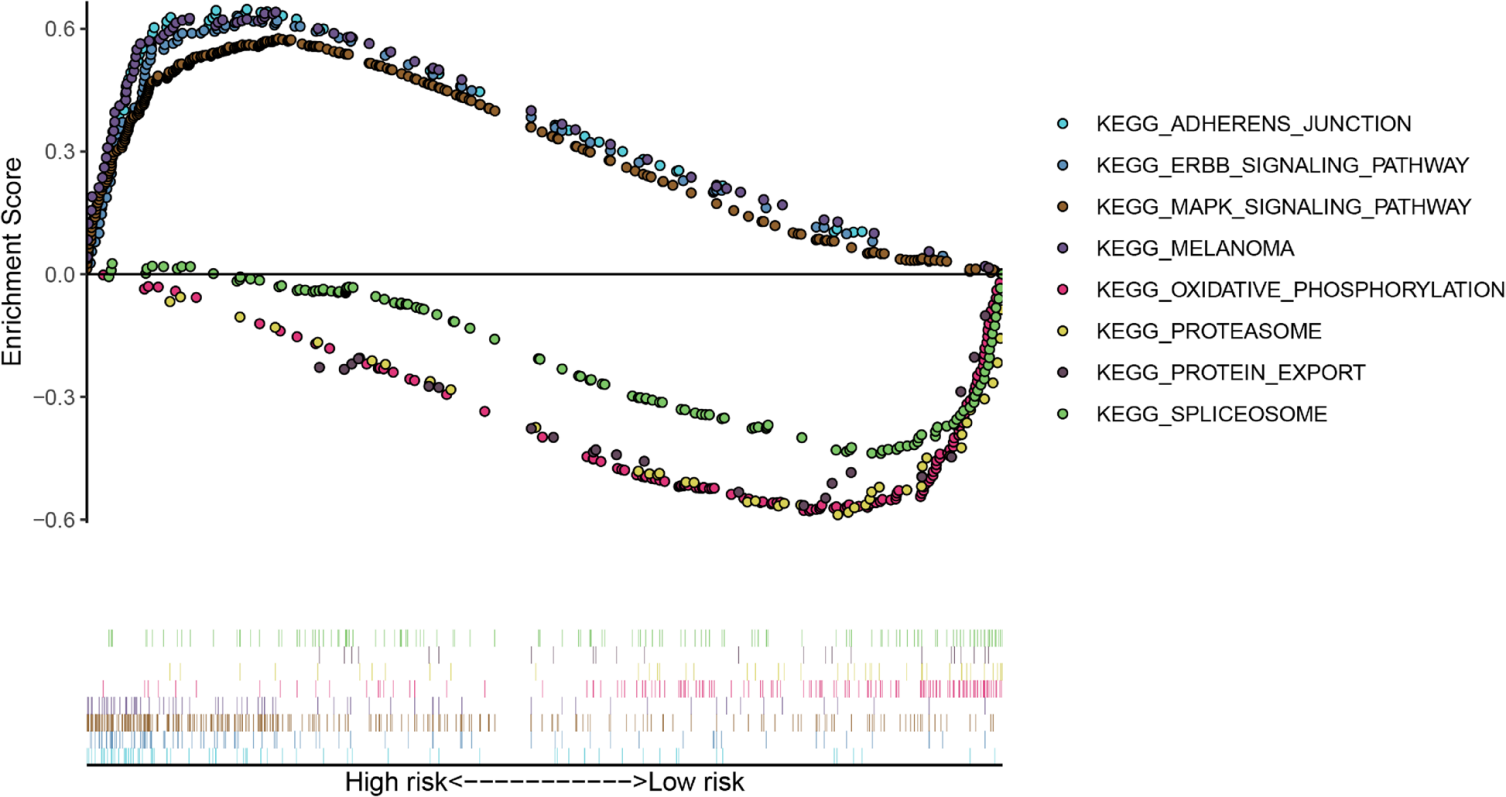
Gene set enrichment analysis (GSEA) between high-risk and low-risk groups based on the prediction model

PCA was used to visualize patient distribution based on a genome-wide expression set, IRG expression set, IR-lncRNA expression set and 6-lncRNA signature. According to the genome-wide and IRG expression sets, PCA showed no significant separation between the groups (Fig. 6A-B). Based on the IR-lncRNA expression set, patients in the low-risk and high-risk groups tended to be divided into two groups (Fig. 6C). However, with the 6-lncRNA signature, patients in the low-risk group and the high-risk group clearly showed different distribution directions (Fig. 6D). We also analysed the relationship between the 6-lncRNA signature and immune cell infiltration and found a positive correlation between macrophages and the risk score (Fig. 6E). GSEA was used to further explore the biological function of the 6-lncRNA signature. GSEA showed that the 6-lncRNA signature in the high-risk group was highly expressed mainly in cell adhesion, the mitogen-activated protein kinase (MAPK) signalling pathway, various cancer-related pathways and the ErbB receptor-related signalling pathway. In the low-risk group, more were associated with spliceosomes and proteasomes, which are involved in the cell cycle, regulate apoptosis and enhance the immune response (Fig. 7).

### Expression of the 6-lncRNA and immune checkpoint-related genes

**Fig. 8:**
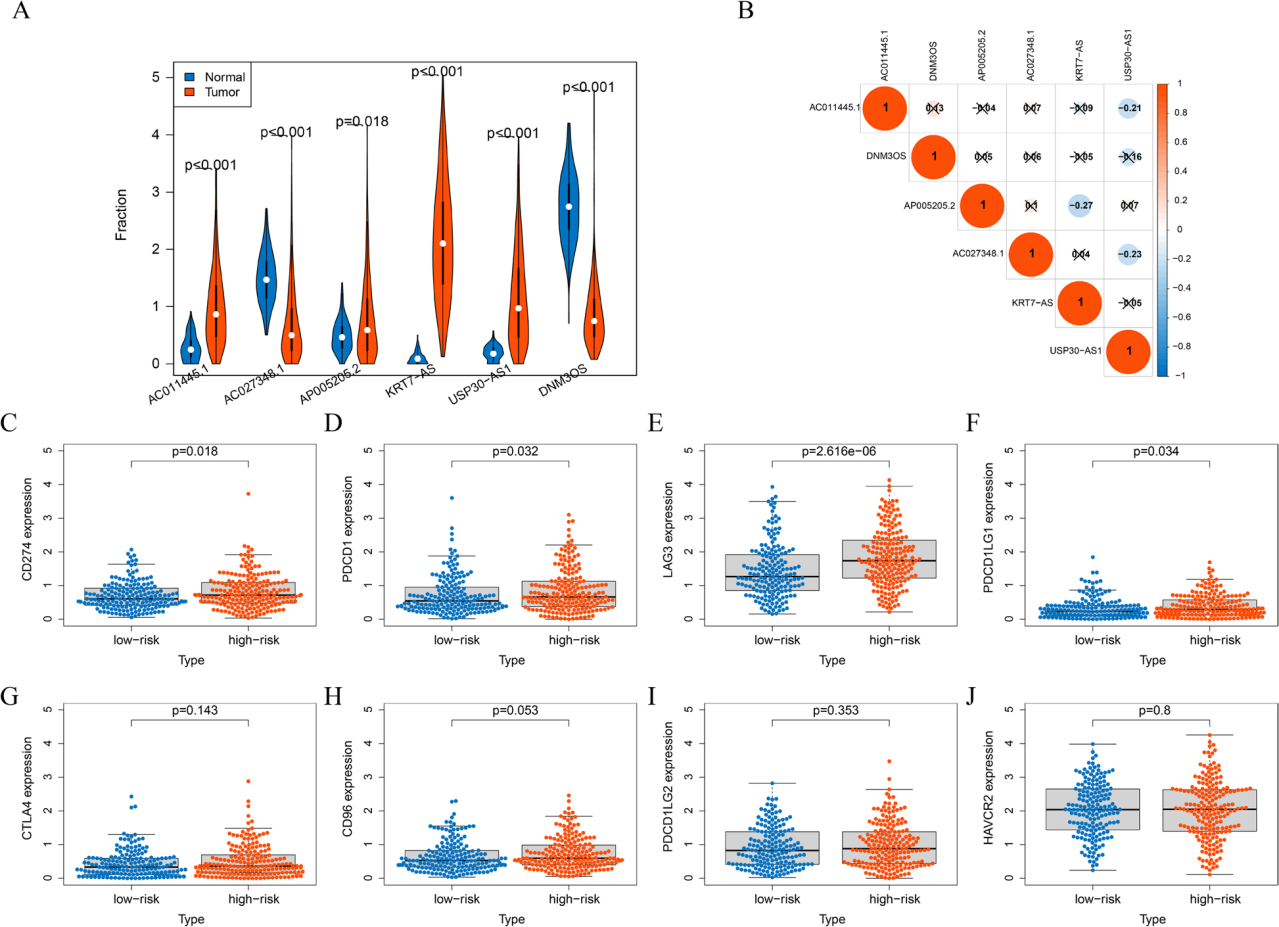
Expression of 6 lncRNAs and immune checkpoint-related genes. **A** The expression levels of 6 kinds of lncRNAs in normal ovarian tissues and ovarian cancer tissues. **B** The Pearson correlation among 6 kinds of lncRNAs. Box plots visualizing the correlation between the risk score and the immune checkpoint-related genes CD274 (**C**), PDCD1 (**D**), LAG3 (**E**), PDCD1LG1 (**F**), CTLA-4 (**G**), CD96 (**H**), PDCD1LG2 (**I**) and HAVCR2 (J)

We compared the expression levels of six lncRNAs (KRT7-AS, USP30-AS1, AC011445.1, AP005205.2, DNM3OS and AC027348.1) in normal ovarian tissues and OC tissues (Fig. 8A). To further understand the interaction between the six lncRNAs, we analysed their expression correlations (Fig. 8B). At the same time, eight immune checkpoint-related genes, including PDCD1 (PD-1), CD274 (PD-L1), PDCD1LG1, PDCD1LG2, CTLA-4, HAVCR2, LAG-3 and CD96, were selected and their expression differences between the high-risk and low-risk groups were analysed. We found that there were four upregulated immune checkpoint genes in the high-risk group with a 6-lncRNA signature, namely, CD274, PDCD1, LAG-3 and PDCD1LG1 (Fig. 8C-J).

## Discussion

EOC has insidious onset, early metastasis and a high recurrence rate. Platinum-based chemotherapy plays an important role in EOC drug treatment, and it is very common for EOC patients with initial platinum sensitivity to develop cancer recurrence and platinum resistance [24]. OzeşAR et al. found that the lncRNA HOTAIR extended NF-κB activity by downregulating I-κB α (NF-κB inhibitor), which affected cellular senescence and platinum resistance in OC [25]. The use of targeted therapies has led to significant changes in the treatment model of EOC, extending the survival of EOC patients and bringing new hopes. Targeted therapy for EOC refers to the use of small molecule compounds or monoclonal antibodies to specifically interfere with the molecular targets of tumour cells to achieve antitumour effects [26]. The efficacy of targeted therapy is closely related to the mutation status of relevant genes, and the occurrence of gene mutations is probabilistic, thus the efficacy of targeted therapy is limited to some extent [27]. Studies have shown that lncRNAs play an important role in the occurrence, development, metastasis, invasion and other biological behaviours of EOC, and their expression is dysregulated in cancer tissues, showing potential as emerging tumour markers [16, 28]. Emerging evidence suggests that lncRNAs play a regulatory role in controlling cancer immunity [10]. For example, the lncRNA NKILA promotes tumour immune escape by sensitizing T cells to activation-induced cell death [29].

At present, IR-lncR prognostic signatures have been reported in lung cancer, cervical cancer, breast cancer and other cancers [30–32]. However, previous studies on the prognostic biomarkers of EOC have mainly focused on mRNAs and microRNAs, and relatively few studies have focused on the prognostic value of IR-lncRs in EOC [33, 34]. Therefore, to better evaluate the immune status and prognosis of EOC patients, we focused on IR-lncRs and developed a risk scoring model in EOC patients based on IR-lncRs for the first time.

In this study, we integrated the gene profiles of the TCGA and GTEx, combined with IRGs from ImmPort, and obtained 421 differentially expressed IR-lncRs through coexpression analysis. Univariate and multivariate Cox regression analyses were performed on the differentially expressed IR-lncRs of 236 EOC patients in the training set. Finally, a 6-lncRNA signature (KRT7-AS, USP30-AS1, AC011445.1, AP005205.2, DNM3OS, and AC027348.1) was determined, which could classify EOC patients into high-risk and low-risk groups, with a significant difference in OS between the two groups (*P* < 0.001). At the same time, we verified the signature in the validation set and the combination set, and the results showed that the 6-lncRNA signature had good predictive ability. Independent prognostic analysis confirmed that the 6-lncRNA signature was superior to other clinicopathologic features in predicting survival.

Some genes in the 6-lncRNA signature have been previously confirmed to play an important role in cancer regulation. Huang et al. found that the expression level of KRT7-AS in gastric cancer cell lines and tissues was significantly higher than that in normal cells and normal adjacent tissues and that KRT7-AS was involved in the pathophysiological process of gastric cancer as a positive regulator of KRT7. High expression of KRT7-AS stimulates cell proliferation, accelerates cell entry into S phase and promotes cell migration [35]. Chen et al. found that KRT7-AS/KRT7 acts as a downstream signalling molecule of mRNA N6-methyladenosine to regulate the lung metastasis of breast cancer cells [36]. Other studies have found that KRT7-AS is associated with advanced stage N colorectal cancer. *Fusobacterium nucleatum* infection promotes the migration of cancer cells in vitro and in vivo in a KRT7-AS-dependent manner [37]. At present, it is not clear whether KRT7-AS is directly involved in the occurrence and development of OC, but it has been reported that KRT7 may promote EMT of OC through the TGF-β/SMad2/3 signalling pathway, and KRT7-AS regulates the expression of KRT7, suggesting that there are numerous links between KRT7-AS and OC [38]. USP30-AS1 is a newly discovered lncRNA transcribed from the antisense chain of the USP30 gene, which is a novel mitochondrial deubiquitinase involved in the regulation of p53 stability and a variety of pathophysiological processes [39]. The regulatory role of USP30-AS1 in cancer has not been thoroughly studied. It has been reported that USP30-AS1 may be associated with cervical cancer, glioblastoma multiforme and bladder cancer, but this is mostly based on bioinformatics analysis [31, 40, 41]. In our study, USP30-AS1 was considered a tumour suppressor, which may require future in vivo and in vitro experiments to reveal the relationship between USP30-AS1 and cancer. Zhang et al. found that DNM3OS was involved in DNA damage repair in oesophageal squamous cell carcinoma after radiation and proposed that DNM3OS might be a target for improving the sensitivity of oesophageal squamous cell carcinoma to radiotherapy [42]. In addition, studies have reported that DNM3OS is associated with poor prognosis of gastrointestinal stromal tumours and liver cancer [43, 44]. Mitra et al. showed that DNM3OS was related to the EMT of OC. After the DNM3OS gene was knocked out in OC cells, RNA sequencing and pathway analysis of differentially expressed genes revealed that multiple EMT-linked pathways were affected, the expression of EMT-related proteins in OC cells was reduced, and migration and invasion were inhibited [2].

Immune cell infiltration in the TME plays a key role in tumorigenesis and progression and affects the clinical prognosis of cancer patients [45]. Macrophages, as important components of the TME, have the ability to inhibit T cell recruitment and function as well as other aspects of tumour immunity and are associated with adverse disease outcomes [46]. In this study, there was a positive correlation between macrophages and the risk score, which we speculated might be related to the poor prognosis of patients in the high-risk group at the level of tumour immunity. In addition, immune checkpoint inhibitors (ICBs), as emerging anticancer targets, have been approved for a variety of malignancies, which may change the treatment model of EOC in the future. In the 6-lncRNA signature that we constructed, the CD274, PDCD1, LAG-3 and PDCD1LG1 genes in the high-risk group were highly expressed, which may have implications for the selection of immunotherapy targets and populations in EOC. However, there are some limitations in our study. First, our signature was only validated internally and not further validated with other external data. Second, it is necessary to further study the functions and mechanisms of these six IR-lncRNAs in combination with basic experiments. In addition, a larger sample size is needed to verify the accuracy of the 6-lncRNA signature in the future.

## Conclusions

In summary, an immune-related lncRNA prognostic evaluation model for EOC was established, which consisted of six lncRNAs (KRT7-AS, USP30-AS1, AC011445.1, AP005205.2, DNM3OS and AC027348.1). The results showed that the model is reliable in predicting the prognosis of clinical patients. We expect that this model will provide ideas for the development of new biomarkers and guide the individualized treatment of patients with EOC.

## Supplementary Information

**Additional file 1.** Supplementary Table S1: A list of 2483 immune-related genes from ImmPort.

**Additional file 2.** Supplementary Table S2: A list of 421 differentially expressed immune-related lncRNAs.

## Data Availability

The datasets generated and analyzed during this study are available in the TCGA, GTEx, ImmPort and Timer database.

## Abbreviations

AUC: Area under the curve
DEGs: Differentially expressed genes
DE-IRGs: Differentially expressed IRGs
EMT: Epithelial mesenchymal transformation
EOC: Epithelial ovarian cancer
GSEA: Gene Set Enrichment Analysis
GTEx: Genotype-Tissue Expression
ICBs: Immune checkpoint inhibitors
ImmPort: Immunology Database and Analysis Portal
IRGs: Immune-related genes
IR-lncRs: Immune-related long noncoding RNAs
LASSO: Least absolute shrinkage and selection operator
LncRNA: Long noncoding RNA
OC: Ovarian cancer
OS: Overall survival
PCA: Principal component analysis
ROC: Receiver operating characteristic
TCGA: The Cancer Genome Atlas
Timer: Tumour Immune Estimation Resource
TME: Tumour microenvironment
